# Early childhood sleep quality in a pediatric cohort: sex-specific differences

**DOI:** 10.3389/frsle.2025.1681175

**Published:** 2025-12-11

**Authors:** Sonali Bose, Kristie R. Ross, Xin Zheng, Naim Xhani, Tamás Ötvös, Francheska M. Merced-Nieves, Susan Redline, Rosalind J. Wright

**Affiliations:** 1 Division of Pulmonary, Critical Care and Sleep Medicine, Department of Medicine, Icahn School of Medicine at Mount Sinai, New York, NY, United States; 2 Department of Pediatrics, Icahn School of Medicine at Mount Sinai, New York, NY, United States; 3 Department of Pediatrics, University Hospitals Rainbow Babies & Children's Hospital, Cleveland, OH, United States; 4 Division of Pediatric Pulmonology and Sleep Medicine, Case Western Reserve University School of Medicine, Cleveland, OH, United States; 5 Departments of Public Health and Environmental Medicine, Icahn School of Medicine at Mount Sinai, New York, NY, United States; 6 Division of Pulmonary, Miller School of Medicine, Critical Care and Sleep Medicine, University of Miami, Miami, FL, United States; 7 Division of Sleep and Circadian Disorders, Brigham and Women's Hospital, Harvard Medical School, Boston, MA, United States

**Keywords:** sleep, actigraphy, polysomnography, sex, child

## Abstract

**Background:**

Poor sleep health in childhood has significant implications for life-long physical, cognitive, and behavioral outcomes, but sleep problems and potential developmentally-driven sex differences in early childhood are poorly understood.

**Objective:**

To objectively assess sleep health and investigate sex-specific differences in sleep in early childhood.

**Methods:**

Three hundred thirty-six children enrolled in the PRogramming of Intergenerational Stress Mechanisms (PRISM) pregnancy cohort in Boston and New York City underwent actigraphy for 7 days. A subset (*n* = 117) completed home unattended Type II polysomnography (PSG). Sleep parameters from actigraphy and PSG were examined using standard descriptive analyses, and non-parametric tests of comparison of means, specifically two-sample Wilcoxon Rank Sum tests, were used to assess differences between boys and girls.

**Results:**

Children were median (IQR) age of 6.9 (4.9, 8.9) years, half were male, and most were Black (46.2%) and/or Hispanic (33.9%). On actigraphy, average sleep duration and sleep efficiency were low compared to published normative data, with median (IQR) time spent asleep of 7.8 (7.4, 8.3) hours and sleep efficiency of 79.1% (74.7, 82.6). Wake duration after sleep onset (WASO) assessed by actigraphy was elevated (median (IQR) of 1.8 (1.4, 2.2) hours), and significantly worse among boys compared to girls (*p* = 0.0007). Overall, boys had significantly more restless and fragmented sleep as measured by both actigraphy and PSG. Sleep disordered breathing events were infrequent, with median (IQR) Apnea-Hypopnea Index of 0.7 (0.3, 1.3) events per hour.

**Conclusion:**

Clinically significant and objectively assessed sleep disturbance was common in this pediatric cohort, and worse in boys compared to girls. These findings emphasize early sex-based sleep disparities warranting intervention to intercept lifelong consequences of poor sleep in early childhood.

## Introduction

Sleep problems are a major pediatric concern affecting 15–50% of young children (Owens, 2008; Blader et al., 1997; Archbold et al., 2002). Adequate sleep is important for normal growth and development; persistent childhood sleep problems impact behavioral (Sivertsen et al., 2015; Hysing et al., 2016), cognitive (Touchette et al., 2007; Seegers et al., 2016), and physical outcomes (Chaput et al., 2016; Gozal et al., 2008), with potential lifelong consequences (Wong et al., 2009; Medic et al., 2017). Optimal sleep health is not just the absence of frank sleep disorders such as obstructive sleep apnea (OSA), but includes broader, multidimensional aspects such as sleep duration and quality (Chaput, 2019). Notably, insufficient or fragmented sleep contributes to poor health outcomes in pre-school and school-age children (Marcus et al., 2013). While treating childhood sleep disorders improves some outcomes (Marcus et al., 2013; Mindell et al., 2006), response to treatment is often incomplete (Chervin et al., 2006; Biggs et al., 2014), suggesting there may be an early critical window during which child sleep problems become trait-like and are more likely to have lasting health impacts. Hence, early identification of suboptimal childhood sleep is paramount to preventing short- and long-term consequences.

However, existing knowledge of early childhood sleep has unfortunately relied on caregiver report–and in some cases, accelerometry-based monitoring—which may result in misclassification of sleep patterns. In contrast, polysomnography (PSG) is considered the gold standard for assessing neurophysiological sleep (including sleep stage distributions, fragmentation, and sleep-disordered breathing). However, PSG in young children is challenging due to logistical hurdles associated with obtaining in-lab sleep studies and the absence of ambulatory protocols to monitor sleep in a child's naturalistic environment. Therefore, the lack of robust objective assessments has limited our understanding of real-world sleep quality of young children.

Furthermore, sleep is a neurodevelopmental phenomenon (Uchitel et al., 2022), and foundational development of neural architecture of sleep begins *in utero* and continues over early childhood. Biological sex influences multiple aspects of brain development and leads to structural and functional dimorphisms in the brain (Uchitel et al., 2022; Cahill, 2006), and sex-based differences in early life brain maturation and plasticity have been postulated to influence sleep. Some studies in healthy newborns have revealed delayed maturation, as well as decreased sleep times and more wakefulness after sleep onset, in boys compared to girls (Franco et al., 2020). This vulnerability is consistent with other developmental trajectories whereby male offspring are more susceptible to early environmental exposures, resulting in higher odds of downstream adverse outcomes. However, studies examining sex differences on sleep patterns have largely been in older children/adolescents and cohorts with co-morbidities (Price et al., 2023; James et al., 2020; Giddens et al., 2022; Elkhatib Smidt et al., 2021; Landeo-Gutierrez et al., 2024), rather than young, otherwise healthy children. To our knowledge, no prior studies have utilized robust objective methods to examine whether sex-based susceptibilities, potentially originating from diverging developmental trajectories, are present during early childhood. Identifying differential sex-based vulnerability in young children is essential to inform early, personalized interventions to prevent life-long consequences of poor-quality sleep.

To address these gaps, we aimed to describe sleep patterns in a pediatric cohort using both actigraphy and unattended in-home PSG, providing objective and comprehensive evaluation within the home environment. Given known sexual dimorphisms in neurodevelopment and the dependence of early neural growth pathways on sleep maturation, we investigated if there were sex differences in observed sleep parameters.

## Materials and methods

### Study population

Participants were from the PRogramming of Intergenerational Stress Mechanisms (PRISM) pregnancy cohort, originally designed to examine associations between perinatal environmental exposures and child respiratory outcomes. PRISM recruited women (*n* = 944) receiving prenatal care from Beth Israel Deaconess Medical Center and East Boston Neighborhood Health Center, Boston, MA (March 2011–December 2013) and Mount Sinai Hospital, New York City, NY (April 2013–February 2020). Eligible women were English- or Spanish-speaking, ≥18 years old, and carrying a single gestation pregnancy; ineligibility criteria included maternal endorsement of ≥7 alcoholic drinks consumed per week prior to pregnancy or any prenatal alcohol use that could impact primary outcomes. Procedures were approved by relevant Institutional Review Boards (IRBs), and mothers provided written consent in their primary language (English or Spanish).

Supplemental funding was secured to assess sleep in children at least 4 years of age over the funding cycle. Enrollment into this sub-study excluded children if he/she: 1. had history of a known genetic, craniofacial upper airway anatomical abnormality, neurologic, pervasive developmental, or psychiatric condition likely to affect the airway, cognition or behavior that could significantly influence the child's ability to comply with the protocol; 2. was using neuro-stimulant medications treating severe behavioral abnormalities; and 3. was using medications treating insomnia or having sedating effects. Caregivers provided written consent, assent was obtained for children over 7 years old, and all children were permitted to refuse any part of the protocol. Caregivers were told to maintain the child's usual sleep schedule and bedtime activities during monitoring. Analyses presented here represents data collected between August 1, 2019 and March 20, 2023.

### Sleep outcomes

#### Accelerometry

Child sleep parameters were objectively assessed using GT3x+ accelerometers (ActiGraph, Pensacola, FL) placed on the child's non-dominant wrist by trained study staff in the home. Participants were asked to wear the accelerometer consecutively for 7 days, and caregivers asked to record sleep diaries. During the COVID-19 pandemic, in efforts to minimize in-person encounters, we developed protocols allowing for either in-person or remote, caregiver accelerometer placement. For remote assessments, kits containing accelerometer devices, adjustable wrist bands, and a positioning tool to ensure that wristbands were placed with appropriate skin apposition were mailed to participants through a tracked parcel service, along with a step-by-step instructional video demonstrating proper device placement. After kits were received, caregivers were guided in real-time over video conference on device placement on the child's wrist, and staff visually confirmed correct placement. All videos and written materials were available in English and Spanish.

Mailed or in-person placed accelerometer devices were either retrieved in person or sent back by the caregiver using a tracked parcel service with a prepaid label. Data were downloaded and, to be as inclusive as possible within an understudied population, recordings with a minimum of 1 overnight period were electronically securely transmitted to the Sleep Reading Center (SRC), Brigham and Women's Hospital, Boston, MA, for annotation and quality control. Main sleep periods were identified using a hierarchical approach that incorporated information from the sleep diary, light markers, and activity counts. Using accelerometry software ActiLife v6.13.6 (Actigraph, Pensacola, FL), the Sadeh sleep algorithm (Sadeh et al., 1994) classified each 60 s epoch as an awake or asleep period. Daily measures were averaged to provide a single estimate. Naps were scored by the same algorithm if reported in diaries and a corresponding activity drop was observed; however, naps were rare and not included in analyses. Adherence to actigraphy was objectively indicated by the presence of motion captured by the accelerometer; periods when the watch was unworn (e.g., if the participant removed it) were not included in analyses.

#### Polysomnography

One night of PSG was performed in a subset of children, either simultaneous to accelerometry or during a different night. Trained, two-person teams set up in-home equipment just prior to the child's regular bedtime involving a portable, configurable system (Bioradio, Great Lakes Neurotechnologies, Cleveland, OH) adapted to collect PSG data in the home environment. Staff placed leads and monitors in a standardized fashion on the scalp and body per American Academy of Sleep Medicine (AASM) guidelines (American Academy of Sleep Medicine, 2016), capturing simultaneous tracings across a full montage of channels, including: left (L) and right (R) electrooculograms (EOG); electroencephalogram (EEG) positions F3, F4, C3, C4, O1, and O2; electromyogram (EMG); airflow through a disposable pediatric sensor (DyMedix Diagnostics Inc, Shoreview, MN); chest and abdominal belts, and EKG leads (Supplementary Figure 2). Monitors were enclosed in a zippered fabric pouch placed at bedside with all connecting wires enclosed in a protective sheath and disposable cover for hygienic purposes. Overnight data collection was unattended, and staff assisted in removing equipment the following morning. Recordings ended when the child, caregiver, or research staff disconnected leads. After onset of the COVID-19 pandemic, study staff wore personal protective equipment (PPE) during all in-person encounters, while continuing the same protocol.

#### Polysomnography (PSG) data management

Data acquired were subsequently downloaded into Biocapture software (Great Lakes Neurotechnologies, Cleveland, OH), exported into raw ASCII format, converted into European Data Format (EDF) and imported into Embla^®^ RemLogic^TM^ PSG Software (version 1.3, Natus Medical, Middleton, WI). A trained sleep physician visually assessed all tracings for sufficient capture of interpretable data and absence of artifacts. A minimum of 4 h of total sleep time recorded was considered acceptable for scoring and diagnostic interpretation, as done in prior studies (Tan et al., 2015). A study was deemed unsatisfactory if: it did not meet at least 4 h of recording time, as studies fewer than 4 h of quality data may not reflect a representative sample of sleep architecture and respiratory events and are therefore considered technically limited or inconclusive; if any key channel (including EEG, EOG, EMG, airflow belt, pulse oximetry, or airflow channels) recorded for less than 4 h; or if there were substantial signal artifacts. Unsatisfactory studies were repeated if caregiver and child agreed. Successful studies were electronically securely transmitted as de-identified EDF files to the SRC. Polysomnographic tracings were analyzed using Profusion PSG 3 (Compumedics, Charlotte, NC) with sleep staging, arousal detection and annotation of apneas and hypopneas manually scored following AASM Manual for the Scoring of Sleep and Associated Events Version 2.2). PSG data quality was assessed by a standardized ordinal scale, with scores ranging from 1, representing a failed study with insufficient hours of usable data, and 6, with all signals good for ≥ 6 h (Supplementary Figure 1). To identify the sleep period, lights-out was set at the epoch just prior to the first epoch of sleep and lights-on was set at the beginning of the epoch following the last epoch of sleep. Assessments captured all sleep stages, including rapid eye movement (REM) and non-REM sleep and respiratory events.

#### Outcome variables

Using prior approaches (Zhang et al., 2024), we assessed standard sleep parameters reflecting both sleep quantity and quality. For accelerometry (reported as averages across days recorded) and polysomnography (single night), primary outcomes were: total nocturnal sleep time (TST) (defined as the number of minutes the child was asleep for their nocturnal sleep period); and sleep efficiency (proportion of the time spent asleep, calculated as percentage of TST/time in bed). Secondary outcomes included: sleep latency (SL); wake after sleep onset (WASO) (minutes inclusive of epochs scored awake after sleep onset); sleep fragmentation index (sum of the movement index scored as epochs with activity divided by total hours in bed × 100); fragmentation index (scored as the percentage of 1-min periods of sleep to total periods of sleep in the sleep period); and movement indices. Additional PSG metrics included: percentage spent in each sleep stage; arousal index (numbers of arousals per hour of sleep); and apnea-hypopnea index (number of apneas plus hypopneas per hour of sleep; hypopneas defined as 50% reductions in airflow for a minimum of 2 breaths associated with a 3% desaturation or arousal).

#### Covariates

Demographic and health-related data was collected during an initial prenatal parent cohort study visit. Height and weight were either recorded at the home visit by staff using a portable stadiometer and scale, or if accelerometry was remote, based on caregiver report or electronic medical record within the prior 6-months.

#### Statistical analyses

Data from completed accelerometry and PSG studies were summarized using descriptive analyses including means, medians, and proportions as appropriate; Wilcoxon Rank Sum Tests were employed for comparisons of distributions across sex. We also analyzed sleep variables by sex individually within subgroups by age, including preschool (4–5 years) and school age (6–11 years), per established AASM pediatric cut-offs (Berry et al., 2020). All analyses were performed using Stata (College Station, TX, version 18).

## Results

### Participant demographics

Three hundred and thirty-six children completed at least 1 night of accelerometry successfully. Median (IQR) age was 6.9 (4.9, 8.9) years at the time of sleep assessment and approximately half (49%) were male. There was no significant difference in age across sexes (*p* = 0.86). Almost all children (*n* = 308, 92%) were born full-term (gestational age ≥ 37 weeks) (Table 1).

**Table 1:** Participant characteristics.

**Characteristic**	**All children (*n* = 336)**
Age (years; median, IQR)	6.9 (4.9, 8.9)
Sex (*n*, % male)	166 (49.4)
BMI-for-age percentile (median, IQR)^a^	71.0 (37.3, 90.2)
Maternal education (*n*, % HS education or less)	118 (35.5)
Race/ethnicity (*n*, %)	
Non-Hispanic White	46 (14.2)
Black/Hispanic Black	150 (46.2)
Non-Black Hispanic	110 (33.9)
Other	19 (5.9)

^a^*n* = 182 (% missing: 46%).

### Accelerometry

Median (IQR) number of 24-h periods with complete accelerometry data was 7(6,8), reflecting a high level of wear compliance, and did not differ by sex (Table 2). Overall, median (IQR) nocturnal sleep duration was 9.7 (9.2, 10.2) hours and not significantly different across boys and girls (z = −0.905, *p* = 0.37). Median (IQR) sleep efficiency was similar across sexes, although statistically higher in girls than boys (80.8 vs. 77.7%, respectively, *z* = 3.482, *p* = 0.0005). In contrast, WASO was quite high across all children (median (IQR) 1.8 (1.4, 2.2) hours), but higher among boys compared to girls (1.9 vs. 1.6 h, respectively, z = −3.407, *p* = 0.0007). Boys were also significantly more restless and had more fragmented sleep compared to girls, with total sleep fragmentation index of 33.1 vs. 30.5%, respectively (z = −2.977, *p* = 0.003) (Table 2). On average, data was similar during weekdays compared to weekends (Supplementary Table 1), and hence no weighting for day of week was performed.

**Table 2:** Sleep parameters obtained through actigraphy (*n* = 336).

**Sleep parameter (median, (IQR))**	**All children (*n* = 336)**	**Boys (*n* = 166)**	**Girls (*n* = 170)**	**Z statistic**	***P*-value**
Number of main sleep intervals	7 (6,8)	7 (6, 8)	7 (6, 8)	1.326	0.19
Average sleep onset latency per main sleep (minutes)	17 (13,23)	17 (13, 22)	17 (13, 23)	−0.357	0.72
Average in-bed duration per main sleep (hours)	10.0 (9.5, 10.5)	10.1 (9.6, 10.5)	9.9 (9.4, 10.6)	−0.993	0.32
Average sleep period duration per main sleep (hours)	9.7 (9.1, 10.2)	9.7 (9.2, 10.2)	9.7 (9.1, 10.2)	−0.905	0.37
Average time spent asleep per main sleep (all days) (hours)	7.8 (7.4, 8.3)	7.8 (7.3, 8.3)	8.0 (7.5, 8.4)	2.026	0.04
Average wake duration after sleep onset (WASO) per main sleep (all days) (hours)	1.8 (1.4, 2.2)	1.9 (1.5, 2.4)	1.6 (1.2, 2.1)	−3.407	0.0007
Average sleep efficiency per main sleep (%)	79.1 (74.7, 82.6)	77.7 (73.2, 81.7)	80.8 (76.7, 83.8)	3.482	0.0005
Average sleep maintenance efficiency per main sleep (all days) (%)	82.1 (77.6, 85.3)	80.7 (76, 84.1)	83.1 (79, 86)	3.474	0.0005
Average movement index per main sleep (all days)	18.7 (15.6, 21.4)	19.6 (16.6, 21.9)	17.6 (15.2, 20.4)	−3.584	0.0003
Average fragmentation index per main sleep (all days)	13.8 (10.8, 16.3)	14.1 (11.5, 16.3)	13.3 (10.4, 16.3)	−1.802	0.07
Total sleep fragmentation (sum of movement and fragmentation indices)	31.9 (27.9, 36.9)	33.1 (29, 37.7)	30.5 (26.2, 35.7)	−2.977	0.003
Average number of awakenings per main sleep	25 (22–29.5)	26 (22, 30)	25 (21, 28)	−2.709	0.007
Average mean length of awakenings per main sleep (minutes)	4.2 (3.6, 5)	4.3 (3.7, 5.3)	4 (3.5, 4.8)	−2.180	0.03

### Home polysomnography (PSG)

One hundred and seventeen children from the NYC site also completed 1 night of at-home unattended PSG. Median (IQR) age of children who underwent PSG was 5.9 (4.9, 6.7) years. Median (IQR) study quality was 4 (2, 5), with only 7 (6%) of participants noted to have “failed” the study and excluded from further analyses. There were no differences in study quality between sexes (*z* = 1.093, *p* = 0.28). Across all participants, PSG recorded a median (IQR) of 8.6 (7.4, 9.4) hours of total sleep time and did not differ by sex (Table 3). Sleep efficiency was 95.7% (89.3, 97.5) and consistent with accelerometer data, was statistically worse in boys vs. girls (94.8 vs. 96.4%, z = 2.855, *p* = 0.004), though clinically similar. WASO overall was 23.3 (12, 48) minutes and significantly higher in boys compared to girls (31 vs. 17.5 minutes, z = −2.966, *p* = 0.003). In addition, overall arousal index was 5.3 times an hour, higher in boys than girls, especially during NREM (6.3 vs. 4.8 times an hour, respectively, *z* = −2.508, *p* = 0.01). Median (IQR) Apnea-Hypopnea Index (AHI) was 0.75 (0.3, 1.3) events/hour with 9% of children with an AHI≥2 and was not significantly different between girls vs. boys (*z* = −1.673, *p* = 0.09). Sleep stage distributions were consistent with normative values (Traeger et al., 2005; Pedersen et al., 2023), with 7% spent in Stage 1 sleep, almost half of the time in Stage N2 sleep, and a little over a quarter in Stage N3 (Table 3).

**Table 3:** Sleep parameters obtained through polysomnography (*n* = 110)^a^.

**Sleep architecture median (IQR)**	**All children (*n* = 110)**	**Boys (*n* = 50)**	**Girls (*n* = 60)**	**Z statistic**	***P*-value**
Total time in bed (hours)	9.2 (7.8, 10.1)	9.5 (8.7, 10.2)	8.9 (7.8, 10.0)	−1.64	0.10
Total sleep time (TST) (hours)	8.6 (7.4, 9.4)	8.9 (7.9, 9.3)	8.5 (7.2, 9.4)	−0.985	0.32
Sleep efficiency (%)	95.7 (89.3, 97.5)	94.8 (87.4, 96.2)	96.4 (92.0, 98.3)	2.855	0.004
Sleep maintenance efficiency (%)	95.7 (89.3, 97.5)	94.8 (87.4, 96.2)	96.4 (92.0, 98.3)	2.855	0.004
Rapid eye movement (REM) latency (sleep onset to first REM) (min)	155. (134, 199)	154 (134, 191)	160.5 (130, 204)	0.063	0.95
Wake after sleep onset (WASO) (min)	23.3 (12, 48)	31 (20.5, 63)	17.5 (8, 41.3)	−2.966	0.003
Stage N1 (% TST)	6.0 (4.4, 9.6)	6.8 (4.5, 9.6)	5.9 (4.4, 9.1)	−1.050	0.30
Stage N2 (% TST)	47.1 (43.2, 53.1)	46 (43.3, 50.4)	47.9 (43.2, 54.2)	1.044	0.30
Stage N3-4 (% TST)	26.4 (20.8, 31.5)	25.9 (19.3, 31.3)	26.8 (21.1, 31.6)	0.925	0.36
Rapid eye movement sleep (% TST)	18.3 (14.8, 21.8)	20 (16.6, 23.2)	18.0 (12.9, 20.4)	−1.760	0.08
Overall arousal index (N/h)	5.3 (4.3, 7.0)	6.2 (4.6, 7.3)	5.1 (4.1, 6.4)	−1.976	0.05
Non-REM (NREM) arousal index (N/h)	5.2 (4, 6.3)	6.3 (4.4, 7.9)	4.8 (3.8, 6)	−2.508	0.01
REM arousal index (N/h)	5.7 (3.9, 7.7)	5.6 (4, 7)	6.2 (3.9, 8.5)	1.001	0.32
Apnea-hypopnea index (3%)	0.75 (0.3, 1.3)	1 (0.4, 1.5)	0.6 (0.2, 1.2)	−1.673	0.09

^a^Seven participants were excluded from the original 117 children who completed PSG due to failing sufficient data quality.

For both actigraphy and PSG-based sleep parameters, separate analyses within sleep developmentally based age groups showed similar results, although in some cases did not reach statistical significance due to smaller sample sizes within subgroups (Supplementary Tables 2, 3).

## Discussion

In this cohort of young children, using comprehensive and robust objective methods, we demonstrated significantly poor sleep quality not attributable to OSA, including markedly low sleep efficiency, long periods of WASO and significant sleep fragmentation. Furthermore, we found sex-based differences in sleep quality, such that boys had clinically and statistically significantly longer WASO, and more fragmented sleep, compared to girls. Our results highlight that suboptimal sleep are identifiable as young as 4 years of age, emphasizing the need for early interventions to prevent life-long developmental and health consequences of poor sleep, especially in boys.

Our results are striking when considered in the context of published normative actigraphy data in pediatric populations (Meltzer et al., 2019; Galland et al., 2018; van Kooten et al., 2021; Sahlberg et al., 2018; James et al., 2020). Children in our cohort slept an average of 7.8 h per night, whereas a meta-analysis of normative activity data reported a pooled mean estimate of total sleep time of 8.6 h for children ages 3 to 5 years and 8.2 h for children 6 to 8 years old (Galland et al., 2018). Similarly, accelerometry-based measurement over multiple days showed that sleep efficiency was reduced in our cohort at an average of 79.1% (with half of the cohort experiencing substantially low efficiencies) compared with 86.3% in the meta-analysis (children 3–14y) (Galland et al., 2018). Most notably, in our cohort, children had a mean WASO of almost 2 h in comparison to 55 (43, 68) min in the referenced study. A second meta-analysis with several years of additional primary literature had similar normative values, although data were not stratified by age group (van Kooten et al., 2021). Ultimately, the reasons for the worse sleep observed specifically in our cohort compared to other studies is unclear, and may be due to a variety of factors, including racial and/or ethnic composition, unmeasured environmental factors (e.g., noise, light at night) unique to our urban sites, or unique sleep hygiene or behaviors. However, importantly, the differences in sleep quality we observed appeared to be driven by increased sleep fragmentation and WASO, with normal sleep onset latency and time spent in bed. These distinctions are critical to better understand the nature of sleep disruption to inform future strategies on improving sleep health in young pediatric populations.

To our knowledge, these are the first home-based PSG data to corroborate accelerometer-based findings of sex differences in sleep quality. Previous investigations into sex-based differences during early childhood have been limited and results have been mixed (Elkhatib Smidt et al., 2021; James et al., 2020), in part due to variable methodology. Nevertheless, one study of preschoolers found that parents reported boys were more likely to have shorter sleep periods than girls, driven by earlier awakenings (McDonald et al., 2014). Other studies have similarly relied on self-report of caregivers who may under- or overestimate sleep abnormalities. To overcome this misclassification, some studies have utilized actigraphy to objectively assess child sleep and found that compared to girls, boys had shorter sleep duration, lower sleep efficiency, and more nighttime awakenings (James et al., 2020; Moore et al., 2011; Matthews et al., 2014). However, other authors have used actigraphy in this age group and did not find a sex-based difference, leading to inconsistent findings (James et al., 2020). Therefore, our rigorous PSG-based findings offer significant advantage over prior limited studies by demonstrating sex-based differences in child sleep based on additional objective assessments, as recently recommended (Sadeh, 2011). Accordingly, these new data add to our clinical understanding of boys being at higher risk for the downstream health effects of poor sleep, emphasizing the need for clinical and public health efforts to focus on this vulnerable subpopulation.

Therefore, while the most striking findings of abnormal sleep quality were identified by accelerometry, home PSG sleep data from a single night yielded important information. PSG allowed us to assess sleep disordered breathing events, which were found to be exceedingly low, with no differences between sexes to explain abnormal sleep. Additionally, prior work has suggested that the ability of actigraphy to assess sleep may lead to misclassification of some parameters when comparing males and females due to increased nocturnal motion in boys misclassified as WASO (Short et al., 2012; Guedes et al., 2016), further highlighting the advantage of PSG. Accordingly, we found that males had increased WASO as measured by actigraphy and home PSG, strengthening our sex-based observations and validating the high degree of WASO. Interestingly, the degree of sleep disturbance (i.e., decreased sleep efficiency and increased WASO) found through multi-day actigraphy in the overall cohort was higher than that found by a single night of PSG. This discrepancy may be due to studies not necessarily overlapping on the same night, as well as that actigraphy may be over-estimating wake periods among both sexes, for example in children with movement disorders and Attention-deficit/Hyperactivity disorder. Alternatively, actigraphy data was collected over 7 days vs. a single night of PSG and may be more representative of usual sleep abnormalities. Nevertheless, sex-based differences identified using both modalities reinforce the need to consider clinical health interventions to improve sleep health in young children, suggesting that boys may be particularly vulnerable.

Mechanisms behind sex-based disparities in human sleep health remain poorly understood. Sex differences in sleep have been identified in animal models such as rodents and fruit fly (*Drosophila*) (Elkhatib Smidt et al., 2021), however mechanisms in humans are likely complex and have been understudied. In epidemiologic studies, sex-based disparities in sleep quality have been in part attributed to elements of sleep hygiene. For example, in one study of Australian children aged 4–6 years, boys with an inconsistent bedtime routine on non-school nights had a higher risk of parent-reported sleep disturbance than girls (Uebergang et al., 2017). In another study, excessive internet use among Chinese adolescents was more strongly associated with sleep disturbance in girls vs. boys (Yang et al., 2018). Other studies have explored whether obesity plays a role in sex-based differences observed in sleep disordered breathing (Knutson, 2005). Ultimately, there is insufficient evidence to explain the etiology of sex-based patterns to child sleep disorders, and further work is warranted. Specifically, given prior literature demonstrating heightened male fetal susceptibility to *in utero* toxins altering neurodevelopmental programming (Yi et al., 2022), future studies considering prenatal and early childhood exposure to neurotoxicants and child sleep health should be a priority.

Our study had many strengths. Importantly, our approaches overcame prior limitations of caregiver- or self-report-based sleep by using both accelerometry-based methods as well as novel procedures to replicate a high-quality, full montage multi-channel PSG in a low-touch manner feasible through unexpected pandemic settings. Additionally, data was collected within a child's natural sleep environment instead of a hospital-based lab, especially important for built environments, which have unique co-exposures relevant to sleep health. As there are currently no commercially available monitors which can remotely capture full polysomnographic montages in children, our high methodologic success allows future collection of robust, unattended sleep data, especially in low-resourced settings.

Our work had a few limitations. Accelerometry assesses sleep/wake based on movement and can misclassify state in both directions. Per the intended study design, as PSG assessments were completed in a subset of children over 1 night while actigraphy was over a 7-day consecutive period, and as each modality has distinct methodology, a dedicated comparison was not performed. However, we compared our findings to meta-analyses of other pediatric studies prone to the same potential misclassification and still found substantial differences. In addition, given the majority of our cohort self-reported Black race and/or Hispanic ethnicity, we had insufficient sample size to examine differences across other demographic factors, e.g., race or ethnicity. Sleep behavioral factors may be contributing to our results, but this data was unavailable for the entirety of the cohort. Finally, our cohort collected Actigraphy data across two urban sites and there may be unaccounted for environmental differences between the two cities which influence sleep; however, sleep parameters were similar across sites with the notable exception of sleep fragmentation being higher among New York vs. Boston participants (data not shown), an area of future research.

In conclusion, objectively measured, clinically significant sleep disturbances were common in early childhood and more prevalent among boys.

## Data Availability

The sleep outcome summary parameters generated for this study, absent protected health information (PHI), are available from the authors without undue reservation. Cohort-level data that contain sensitive information can be shared when appropriate permissions and protections are in place.
