# Supplementary material for: Early childhood sleep quality in a pediatric cohort: sex-specific differences
Source: Front Sleep. 2025 Dec 11;4:1681175. doi: 10.3389/frsle.2025.1681175 (PMC12713980; doi:10.3389/frsle.2025.1681175)
Supplement: Supplementary file 1 [file Supplementary_file_1.docx]

**Supplemental tables**

**eTable 1. Sleep parameters comparing weekday and weekend sleep periods**

| **Sleep parameter** | **All days**  **(median, (IQR))** | **Weekday**  **(median, (IQR))** | **Weekend**  **(median, (IQR))** |
| --- | --- | --- | --- |
| Number of main sleep intervals | 7 (7,8) | 5 (5,6) | 2(2,2) |
| Average in-bed time (24-hour time) | 22:08 (21:19, 23:09) | 21:58 (21:07, 23:00) | 22:50 (21:42, 23:55) |
| Average out-of-bed time (24-hour time) | 7:56 (7:14, 8:51) | 7:37 (6:59, 8:38) | 8:31 (7:28, 9:37) |
| Average sleep onset latency per main sleep (minutes) | 17 (13,22) | 16 (13,21) | 16 (12,23) |
| Average sleep period duration per main sleep (hours) | 9.7 (9.1, 10.2) | 9.6 (9.1, 10.3) | 9.8 (9.0, 10.6) |
| Average time spent asleep per main sleep (all days) (hours) | 7.8 (7.4, 8.3) | 7.9 (7.3, 8.4) | 7.9 (7.4, 8.5) |
| Average sleep efficiency per main sleep (%) | 79.1 (74.7, 82.6) | 79.3 (75.0, 82.9) | 79.0 (74.4, 84.1) |
| Total sleep fragmentation (sum of movement and fragmentation indices) | 31.9 (27.9, 36.9) | 31.9 (27.8, 37.8) | 31.8 (25.5, 38.0) |
| Average number of awakenings per main sleep | 25 (22, 29.5) | 25 (22-29) | 25 (20-30) |
| Average mean length of awakenings per main sleep (minutes) | 4.2 (3.6-5.0) | 4.1 (3.5, 5.0) | 4.1 (3.3, 5.1) |

**eTable 2a. Sleep parameters obtained through actigraphy among children aged 4-5 years**

| **Sleep parameter**  **(median, (IQR))** | **All children**  **(n=92)** | **Boys**  **(n=44)** | **Girls**  **(n=48)** | **Z statistic** | **p-value** |
| --- | --- | --- | --- | --- | --- |
| Number of main sleep intervals | 7 (6,8) | 7 (6, 8) | 7 (6, 8) | 0.080 | 0.94 |
| Average sleep onset latency per main sleep (minutes) | 18 (13,24.5) | 17 (14, 22) | 18.5 (13, 26) | 0.513 | 0.61 |
| Average in-bed duration per main sleep (hours) | 10.2 (9.7, 10.9) | 10.5 (9.8, 11) | 10.1 (9.6, 10.8) | -1.571 | 0.12 |
| Average sleep period duration per main sleep (hours) | 9.9 (9.3, 10.5) | 10.1 (9.5, 10.7) | 9.8 (9.3, 10.4) | -1.544 | 0.12 |
| Average time spent asleep per main sleep (all days) (hours) | 8 (7.3, 8.5) | 7.9 (7.3, 8.4) | 8.1 (7.4, 8.6) | 0.911 | 0.36 |
| Average wake duration after sleep onset  . [WASO] per main sleep (all days) (hours) | 1.9 (1.5, 2.5) | 2.2 (1.7, 2.9) | 1.5 (1.8, 2.2) | -2.259 | 0.02 |
| Average sleep efficiency per main sleep (%) | 78.5 (73.7, 81.5) | 75.5 (71.6, 81.1) | 79.5 (75.6, 82) | 1.931 | 0.05 |
| Average sleep maintenance efficiency per main sleep (all days) (%) | 81.0 (76.2, 84.8) | 79.1 (73.7, 83.1) | 82.7 (78.2, 85.4) | 1.982 | 0.05 |
| Average movement index per main sleep (all days) | 19.4 (16.5, 22.2) | 20.4 (17.1, 23) | 18.0 (16.1, 21.0) | -1.841 | 0.07 |
| Average fragmentation index per main sleep (all days) | 15.1 (11.6, 18.2) | 15.6 (12.6, 17.9) | 13.9 (10.8, 18.5) | -0.836 | 0.41 |
| Total sleep fragmentation (sum of movement and fragmentation indices) | 34.35 (28.9, 39.6) | 36.3 (30.6, 40) | 31.5 (28.2, 39.6) | -1.591 | 0.11 |
| Average number of awakenings per main sleep | 25 (21.5-30) | 27 (22, 31) | 24 (20.5, 28.5) | -1.985 | 0.05 |
| Average mean length of awakenings per main sleep (minutes) | 4.6 (3.7, 5.4) | 4.7 (4.0, 5.8) | 4.5 (3.7, 5.2) | -1.091 | 0.28 |

**eTable 2b. Sleep parameters obtained through actigraphy among children aged 6-11 years**

| **Sleep parameter**  **(median, (IQR))** | **All children**  **(n=244)** | **Boys**  **(n=122)** | **Girls**  **(n=122)** | **Z statistic** | **p-value** |
| --- | --- | --- | --- | --- | --- |
| Number of main sleep intervals | 7 (6.5,8) | 7 (7, 8) | 7 (6, 8) | 1.503 | 0.13 |
| Average sleep onset latency per main sleep (minutes) | 17 (13,22) | 17 (13, 23) | 17 (13, 21) | -0.754 | 0.45 |
| Average in-bed duration per main sleep (hours) | 9.9 (9.4, 10.4) | 9.8 (9.3, 10.4) | 9.9 (9.3, 10.4) | -0.370 | 0.71 |
| Average sleep period duration per main sleep (hours) | 9.6 (9.1, 10.1) | 9.6 (9.1, 10.1) | 9.6 (9.0, 10.1) | -0.196 | 0.84 |
| Average time spent asleep per main sleep (all days) (hours) | 7.8 (7.4, 8.3) | 7.7 (7.3, 8.2) | 8.0 (7.5, 8.3) | 1.868 | 0.06 |
| Average wake duration after sleep onset  . [WASO] per main sleep (all days) (hours) | 1.7 (1.4, 2.1) | 1.9 (1.5, 2.3) | 1.6 (1.2, 2.0) | -2.887 | 0.004 |
| Average sleep efficiency per main sleep (%) | 79.6 (75.9, 82.9) | 78.2 (73.8, 81.8) | 81.2 (77, 84.2) | 3.069 | 0.002 |
| Average sleep maintenance efficiency per main sleep (all days) (%) | 82.5 (78.4, 85.3) | 80.9 (76.5, 84.3) | 83.5 (79.7, 86.6) | 3.011 | 0.003 |
| Average movement index per main sleep (all days) | 18.3 (15.5, 21.1) | 19.3 (16.5, 21.7) | 17.4 (14.7, 20.3) | -3.181 | 0.002 |
| Average fragmentation index per main sleep (all days) | 13.5 (10.6, 15.9) | 13.7 (11.1, 15.9) | 13 (10.2, 15.4) | -1.534 | 0.13 |
| Total sleep fragmentation (sum of movement and fragmentation indices) | 31.5 (27.6, 36.2) | 32.6 (28.8, 36.8) | 30.3 (26, 35.4) | -2.677 | 0.007 |
| Average number of awakenings per main sleep | 25 (22-29) | 26 (23, 30) | 25 (21, 27) | -1.998 | 0.05 |
| Average mean length of awakenings per main sleep (minutes) | 4 (3.5, 4.8) | 4.2 (3.7, 5) | 4 (3.4, 4.6) | -2.027 | 0.04 |

**eTable 3a. Sleep parameters obtained through polysomnography, ages 4-5 years**

| **Sleep architecture**  **Median (IQR)** | **All children**  **(n=51)** | **Boys**  **(n=24)** | **Girls**  **(n=27)** | **Z statistic** | **p-value** |
| --- | --- | --- | --- | --- | --- |
| Total time in bed (hours) | 9.2 (8.0, 10.1) | 9.4 (8.8, 10.5) | 8.5 (7.6, 9.5) | -2.699 | 0.007 |
| Total sleep time (hours) | 8.6 (7.6, 9.4) | 8.9 (8.3, 9.6) | 8.4 (7.2, 9.3) | -2.293 | 0.02 |
| Sleep efficiency (%) | 96.1 (94.1, 97.6) | 95.4 (93.5, 97.1) | 96.4 (94.8, 98.5) | 1.699 | 0.09 |
| Sleep maintenance efficiency (%) | 96.1 (94.1, 97.6) | 95.4 (93.5, 97.1) | 96.4 (94.8, 98.5) | 1.699 | 0.09 |
| Rapid eye movement (REM) latency (sleep onset to first REM) (min) | 145 (112, 177) | 145 (121, 177) | 143 (96, 186) | -0.671 | 0.50 |
| Wake after sleep onset (min) | 20.5 (11.5, 33.5) | 26.3 (16, 38.3) | 17 (7.5, 29) | -2.124 | 0.03 |
| Stage N1 (% TST) | 7.6 (4.6, 10.2) | 8.5 (4.8, 10.3) | 7.0 (4.6, 9.8) | -0.691 | 0.49 |
| Stage N2 (% TST) | 46.2 (42.8, 52.2) | 44.8 (41.9, 50.4) | 47.0 (43.2, 52.8) | 0.571 | 0.57 |
| Stage N3-4 (% TST) | 25.1 (17.8, 31.5) | 20 (16.4, 29.2) | 26.8 (21.1, 31.6) | 1.553 | 0.12 |
| Rapid eye movement (REM) sleep (% TST) | 20.1 (14.9, 22.7) | 22 (19.1 24.8) | 18.1 (14, 20.7) | -2.204 | 0.03 |
| Overall arousal index (N/h) | 5.6 (4.8, 7.0) | 5.8 (4.8, 7.3) | 5.5 (4.1, 6.9) | -1.063 | 0.29 |
| NREM Arousal index (N/h) | 5.6 (4.5, 6.6) | 6.3 (4.7, 7) | 5.2 (3.9, 6.6) | -1.403 | 0.16 |
| REM Arousal index (N/h) | 6.1 (4.4, 8.2) | 6.0 (4.8 6.6) | 6.9 (3.8, 10) | 0.451 | 0.66 |
| Apnea-Hypopnea Index (3%) | 1 (0.3, 1.5) | 1.25 (0.6, 1.9) | 0.6 (0.1, 1.2) | -2.322 | 0.02 |

**eTable 3b. Sleep parameters obtained through polysomnography ages 6-11 years**

| **Sleep architecture**  **Median (IQR)** | **All children**  **(n=59)** | **Boys**  **(n=26)** | **Girls**  **(n=33)** | **Z statistic** | **p-value** |
| --- | --- | --- | --- | --- | --- |
| Total time in bed (hours) | 9.5 (7.8, 10.2) | 9.6 (7.6, 10) | 9.4 (7.9, 10.2) | 0.198 | 0.84 |
| Total sleep time (hours) | 8.6 (7.1, 9.4) | 8.4 (7.5, 9.3) | 8.6 (7.2, 9.7) | 0.763 | 0.45 |
| Sleep efficiency (%) | 94.8 (87.4, 97.3) | 92.9 (86.2, 95.6) | 96.4 (88.7, 98.1) | 2.367 | 0.02 |
| Sleep maintenance efficiency (%) | 94.8 (87.4, 97.3) | 92.9 (86.2, 95.6) | 96.4 (88.7, 98.1) | 2.367 | 0.02 |
| REM latency (sleep onset to first REM) (min) | 166 (139, 205) | 166 (137, 205) | 165.5 (147.5, 206) | 0.266 | 0.79 |
| Wake after sleep onset (min) | 30 (12, 68.5) | 37.5 (26, 76.5) | 19.5 (8.5, 52.5) | -2.054 | 0.04 |
| Stage N1 (% TST) | 5.3 (4.4, 7.4) | 5.9 (4.5, 7.4) | 5.1 (4.0, 7.9) | -1.009 | 0.31 |
| Stage N2 (% TST) | 48.0 (44.3, 53.2) | 47.2 (44.3, 50.1) | 50.2 (43.2, 55.1) | 1.071 | 0.28 |
| Stage N3-4 (% TST) | 26.8 (23.5, 31.4) | 26.6 (24.7, 31.4) | 27.2 (21.5, 32.6) | -0.047 | 0.96 |
| Rapid eye movement sleep (% TST) | 18.0 (14.8, 20.4) | 18.1 (15.5, 21) | 17.9 (12.8, 20.4) | -0.375 | 0.71 |
| Overall arousal index (N/h) | 5 (4, 6.8) | 6.3 (3.6, 7.6) | 4.9 (4.1, 5.4) | -1.376 | 0.17 |
| NREM Arousal index (N/h) | 4.9 (3.8, 6.7) | 6.4 (4.1, 8) | 4.6 (3.8, 5.4) | -1.939 | 0.05 |
| REM Arousal index (N/h) | 5.4 (3.7, 7.3) | 4.8 (3.5 7.3) | 5.8 (3.9, 7.3) | 0.970 | 0.33 |
| Apnea-Hypopnea Index (3%) | 0.6 (0.3, 1.2) | 0.55 (0.3, 1.2) | 0.7 (0.3, 1.1) | -0.130 | 0.90 |

**Supplemental figures**

**eFigure 1. Data quality scale. Tracings from each overnight study were evaluated and classified into one of these categories based on the duration and quality of each channel.**

**_1-Failed_** _- less than 4 hours of usable data._

**_2-Fair_** _- At least one frontal or central EEG (F3, F4, C3, C4), one respiratory channel (airflow (*) or either band) usable for minimum of 4 hours lights off to lights on, OR study scored sleep/wake only (because of study- tolerance issues) with usable oximetry for >75% of study duration. May have < 4 hours oximetry._

**_3-Good_** _- At least one frontal or central EEG (F3, F4, C3, C4), one EOG, one respiratory channel (airflow (*) or either band), and oximetry good for 6 hours lights off to lights on and minimum of 50% of the sleep time._

**_4-Very Good_** _- At least one frontal or central EEG (F3, F4, C3, C4), one EOG, airflow, one belt (either thoracic or abdominal), and oximetry good for 6 hours lights off to lights on and minimum of 50% of the sleep time._

**_5-Excellent_** _- At least one frontal or central EEG (F3, F4, C3, C4), one EOG channel, chin, airflow, both belts, and oximetry good for 6 hours lights off to lights on and minimum of 75% of the sleep time._

**_6-Outstanding_** _- All signals good for at least 6 hours lights off to lights on and almost entire (95%) sleep time._

**eFigure 2. Representative tracings from home polysomnography (PSG) setups**. Leads were placed in a standardized fashion on the scalp and body in accordance with American Academic of Sleep Medicine (AASM) guidelines. (A) Simultaneous tracings across a full montage of channels, including the following: left (L) and right (R) electrooculograms (EOG); electroencephalogram (EEG) positions including F3, F4, C3, C4, O1, and O2; electromyogram (EMG); airflow through a disposable pediatric sensor; chest and abdominal belts, and EKG. (B) Example of a tracing in which a central sleep apneic event was identified in the epoch (red bracket).

A)


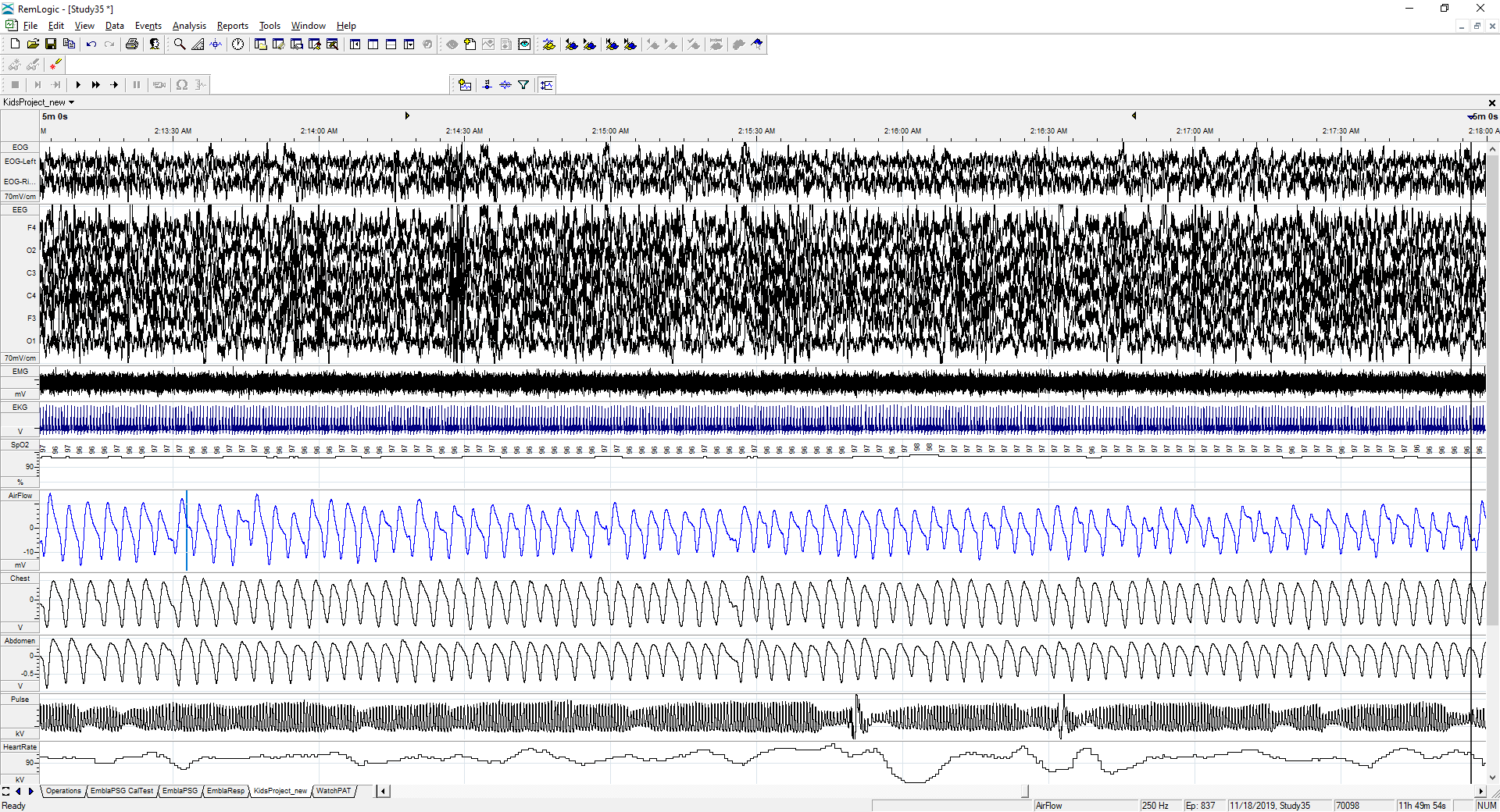


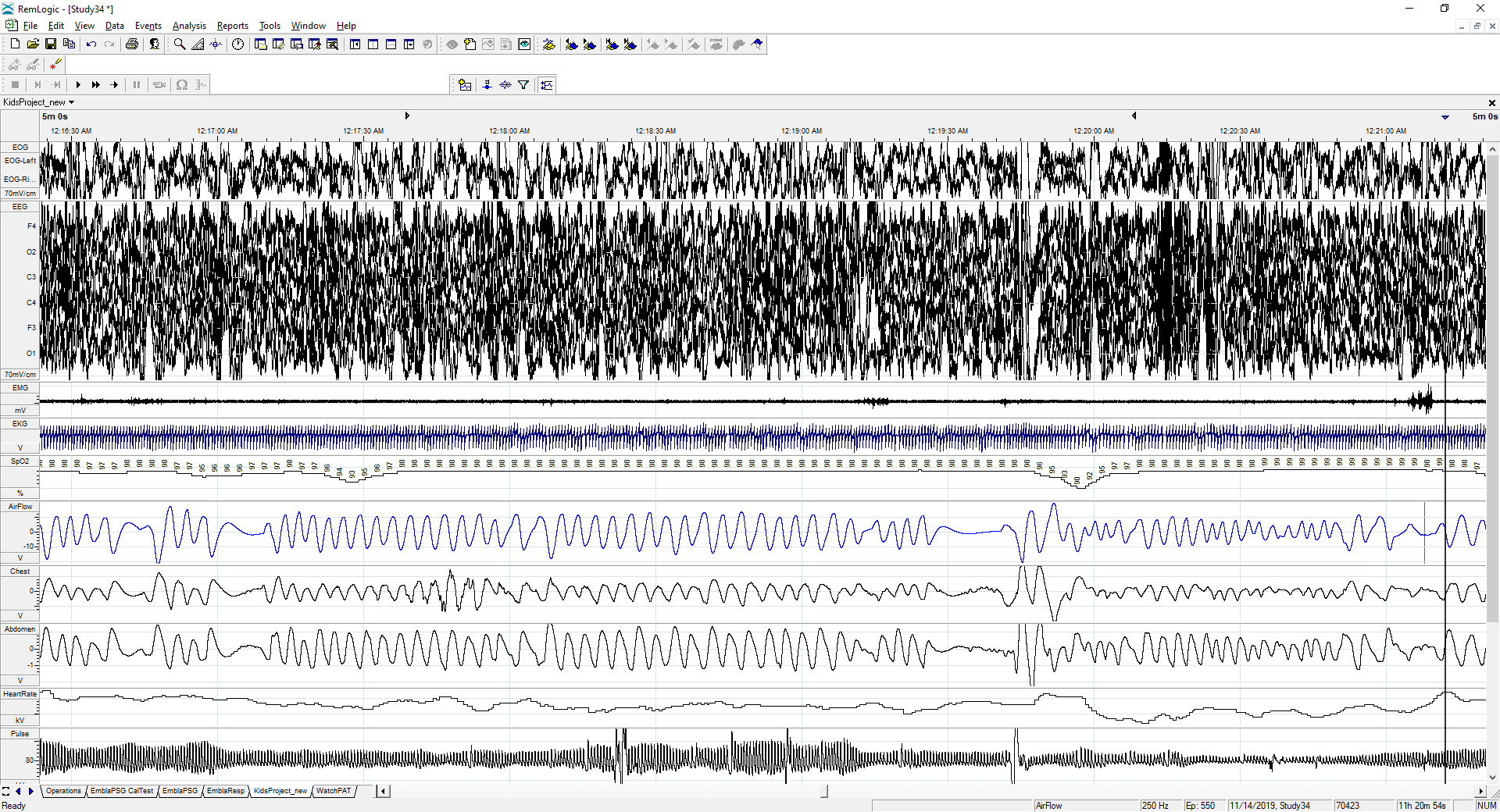


**B)**
